# Effect of Manipulation Methods and Storage Environments on the Microstructural, Chemical, and Mechanical Properties of Calcium-Enriched Mixture Cement

**DOI:** 10.1155/ijbm/5560351

**Published:** 2025-01-20

**Authors:** Leyla Roghanizadeh, Hassan Torabzadeh, Ardavan Parhizkar, Alireza Akbarzadeh Baghban, Saeed Asgary

**Affiliations:** ^1^Iranian Center for Endodontic Research, Research Institute for Dental Sciences, Shahid Beheshti University of Medical Sciences, Tehran 1983963113, Iran; ^2^Dental Research Center, Research Institute for Dental Sciences, Shahid Beheshti University of Medical Sciences, Tehran 1983963113, Iran; ^3^Proteomics Research Center, Department of Biostatistics, School of Allied Medical Sciences, Shahid Beheshti University of Medical Sciences, Tehran 1971653313, Iran

**Keywords:** biomaterials, calcium-enriched mixture cement, energy-dispersive X-ray spectroscopy, Fourier transform infrared spectroscopy, mechanical properties, X-ray diffraction

## Abstract

This study aimed to evaluate the impact of different manipulation methods and storage environments on the microstructural, chemical, and mechanical properties of calcium-enriched mixture (CEM) cement. Four sample groups were examined, including nondried (ND-I) and dried (D-I) groups placed directly in an incubator, dried samples stored in phosphate-buffered saline (PBS) (D-P), and dried samples stored in distilled water (D-W). Various analyses, including Vickers microhardness, compressive strength, Fourier transform infrared spectroscopy (FTIR), X-ray diffraction (XRD), and scanning electron microscopy (SEM) with energy-dispersive X-ray spectroscopy (EDS) were conducted after incubating the samples for 7 days. The data were analyzed by Shapiro–Wilk, Levene, independent *t*, one-way ANOVA, and Tukey HSD tests. Key findings include the ND-I group exhibited a significantly longer setting time but the lowest microhardness and compressive strength. D-P showed the highest microhardness, while D-W displayed the highest compressive strength. FTIR analysis revealed vibration modes related to (PO4)^3−^ ions and Si compounds in all groups, with dried groups showing more vibrations of (PO4)^3−^ ions and OH groups, and D-P and D-W groups displayed vibration modes of (CO3)^2−^ ions. XRD analysis indicated increased tri/dicalcium silicate reflections in CEM groups exposed to PBS or distilled water. D-I and D-W groups presented hexagonal or rectangular cubic and needle-like crystals, while D-P showed a homogeneous globular structure covered with fine crystals. The order of the weight percentage of major elemental constituents of D-P group was oxygen, calcium, phosphorus, zirconium, barium, carbon, silicon, and sulfur. Incremental placement, drying each increment, and exposing CEM to PBS/tissue fluids result in a faster set and more tolerant cement with a more uniform microstructure. The formation of hydroxyapatite can occur on the surface of the set cement.

## 1. Introduction

Calcium silicate–based (CSB) cements are dynamic substances with biological reactions that accelerate the production of mineralized deposits [1]. Mineral trioxide aggregate (MTA) (ProRoot; Dentsply Tulsa Dental, Tulsa, OK, USA) was the first introduced CSB with many successful applications in dentistry/endodontics. In recent years, new types of endodontic biomaterials have been introduced in dentistry such as Biodentine (Septodont, Saint Maur des Fossés, France) and calcium-enriched mixture (CEM) cement (Bionique Dent, Tehran, Iran) [2]. As one of the widely used bioceramics, CEM consists of various calcium compositions. The success of this material has been documented in various methods of vital pulp therapies and other reparative/regenerative treatments [3, 4]. The chemical compositions and therapeutic capabilities of CBS cements can determine the outcome of treatments using these cements. Therefore, knowledge about the nature of these materials and their correct handling and application should always be up-to-date [5]. The kinetics, hydration progress, and physical/mechanical properties of CSB cements are influenced by several factors [6, 7] such as particle size [8], liquid-to-powder ratio [9], condensation pressure [10], placement technique [11], storage environment [12], and storage temperature [6].

Various studies have investigated the properties of CEM cement. Under conditions without cement drying, a setting time of 50–78 min was achieved [13–15]. Moreover, reducing the liquid-to-powder ratio to 0.33 in the cement mixture increases its microhardness [9] and compressive strength [16]. Fourier transform infrared (FTIR) spectroscopy uses scans over infrared wavelengths to provide a better insight into the chemical components and functional groups of the material [17]. As far as we have searched the literature, FTIR has not been performed for this cement. Scanning electron microscopy/energy-dispersive X-ray spectrometry (SEM/EDS) can couple high-resolution microscopic details with a large depth of field to have chemical and microstructural characterization of materials [18]. CEM cement showed the presence of coarse crystalline particles in a matrix of finer amorphous material in SEM analysis [14]. X-ray diffraction (XRD) is used to characterize the position of atoms and their arrangement and allows the identification of multiple minerals present in a sample [19]. The XRD examination of CEM cement revealed the presence of tricalcium silicate, dicalcium silicate, and calcium carbonate [20].

According to the current instructions of the CEM manufacturer, achieving a creamy mixture after mixing powder and liquid is the criterion for the correct consistency and a suitable water content in the cement. However, clinical use has shown that drying the cement during the placement of each part can result in better consistency and favorable mechanical properties. In addition, previous studies showed that different environmental conditions affect the chemical composition and microstructure of CSB cements [20].

This study aimed to evaluate the results of setting time, compressive strength, microhardness, FTIR, SEM, EDS, and XRD on the samples of CEM cement under different methods of placement/manipulation and in various storage environments. The null hypothesis (H0) in this study was that different placement methods and storage environments do not significantly influence the mechanical/chemical/microstructural properties of CEM cement and the results of the performed tests.

## 2. Materials and Methods

This study was approved by the Research Institute for Dental Sciences (RIDS) at Shahid Beheshti University of Medical Sciences. Ethical approval for the study was granted by the Ethics Committee of RIDS (IR.SBMU.DRC.REC.1400.002).

To determine the sample size, in the case of setting time where we have two groups, according to the quantitative nature of the variables, the following formula was used:

(1) $$\begin{array}{c} n=\frac{{\left(\;z_{1-\alpha /2}+z_{1-\beta }\;\right)}^{2}\left(\sigma_{1}^{2}+\sigma_{2}^{2}\;\right)}{{\left(\;{µ}_{1}-{µ}_{2}\right)}^{2}}. \end{array}$$

Based on *α* = 0.05, *β* = 0.2, power = 80%, and means and standard deviations from recent similar papers [13, 21], for the setting time, 9 samples were determined for each group (*n* = 9), and a total of 18 samples were tested. There is no closed mathematical formula to calculate the sample size in tests with 5 groups (microhardness and compressive strength), and the PASS software (PASS Version 2021; NCSS, Kaysville, Utah, USA) performs so many calculations based on the means and standard deviations of the previous similar studies [9, 16, 22, 23], to achieve the appropriate sample size. For microhardness, 7 samples for each group (*n* = 7) and a total of 35 samples were tested, and for compressive strength, 8 samples for each group (*n* = 8) and a total of 40 samples were tested. In recent similar works on CBS cements [24–27], one to five samples from each group were examined for SEM-EDS, FTIR, and XRD tests. In this study, it was decided to use three samples for each of the aforementioned tests based on the average number of samples from the previous studies.

### 2.1. Setting Time

To evaluate the “Properties after Mixing” of the cement, a setting time evaluation was performed. The procedures described in “ISO 9917-1:2007” [28] and “Standard Test Method for Time of Setting of Hydraulic-Cement Paste by Gillmore Needles” [29]. CEM powder and liquid (Bionique Dent, Tehran, Iran) were mixed and prepared according to the manufacturer's instructions. The mixture was then transferred into cylindrical plexiglass molds made of polymethyl methacrylate [30] with an inner diameter of 10 mm and a height of 1 mm.

Two test groups were considered: Group 1 (ND-I), not dried/directly into incubator: The mixture was transferred to the mold in one piece and fitted with a plastic instrument. Group 2 (D-I), dried/directly into incubator: The mixture was transferred to the mold part by part, excess moisture was absorbed by a cotton pellet, and the sample was dried. In both groups, each mold was slightly overfilled and a glass slide was drawn over the filled mold to achieve a smooth surface. A wet gauze was placed on top, and the samples were incubated at 37 ± 1°C and 90% humidity. After 30 min, the samples were taken out for the setting time test.

A 1-mm-diameter flat-end indentation needle from Gilmore apparatus (Taksaz-ideh, Tehran, Iran), weighing 400 g, was gently pulled down perpendicular to the surface of the setting cement at progressive time points. If the indentation was faded and incomplete, the cement was considered as set and the time was recorded. The setting time was calculated from the endpoint of mixing to the time point at which the material was set.

### 2.2. Microhardness

The evaluation of surface microhardness followed the ASTM E384 standard [31]. Stainless steel cylinders with a height of 6 ± 0.1 mm and a diameter of 4 ± 0.1 mm were used as molds. Five groups were considered. Seven samples (*n* = 7) and a total of 35 cylindrical samples were tested in each group. The inner surfaces of the split molds were lubricated with Vaseline [32] using a cotton swab and placed over a glass slab.

• Group 1 (ND-I), not dried: The mixture was transferred into the mold in one piece and fitted with a plastic instrument. A wet gauze was placed in a petri dish, and the mold was positioned on the wet gauze inside the petri dish. The petri dish was stored in an incubator with a temperature of 37 ± 1°C and a humidity of 90% for 7 days.
• Group 2 (D-I), dried/directly to incubator: The cement was transferred part by part into the mold, and excess moisture was absorbed by a cotton pellet. Similar to Group 1, the mold was placed on a wet gauze in a petri dish and stored in the same incubator for 7 days.
• Group 3 (D-P), dried—stored in phosphate-buffered saline (PBS): The mold containing dried CEM was wrapped in a gauze and immersed in a 50-mL polypropylene falcon tube (Kian Tajhizteb, Tehran, Iran) filled up to 20 mL with PBS (Bio Idea, Tehran, Iran). The falcon door was closed, and the tube was placed in the same incubator as Group 1 for 7 days.
• Group 4 (D-W), dried—stored in distilled water: Similar to Group 3, the mold containing dried CEM was wrapped in gauze and immersed in a falcon tube, which was kept in the same incubator for 7 days, but the falcon contained 20 mL of distilled water.
• Groups 5 (D-1/2 PBS), dried—one side in contact with PBS: A gauze was placed in a petri dish and filled with PBS up to 5-mm height. Then, the mold containing dried CEM was placed on the gauze in the petri dish so that only the bottom of the mold came into contact with PBS (later, this side was tested). The petri dish was stored in the same incubator for 7 days.

After 7 days, the molds were removed from the falcons/petri dishes and the samples were removed from the molds. After drying in room air for 5 min, 1200 grit sandpaper was gently used. Vickers hardness test was performed on each sample using a microhardness tester (Zwick/Roell, Indentec ZHVμ-S, West Midlands, UK). A force of 100 g was applied for 10 s using a square pyramid-shaped diamond indenter three times at three widely separated points (3 different readings). Finally, the Vickers microhardness (in kg/mm^2^ unit) was calculated based on the following formula:

(2) $$\begin{array}{c} \text{Hardness}=\frac{2\times F\times \sin\;136{}^{\circ}/2}{d^{2}}=1.854\times \frac{F}{d^{2}}\text{ approximately}. \end{array}$$

### 2.3. Compressive Strength

The compressive strength was conducted to evaluate the cement's “Properties after Setting,” following the procedures outlined in “ISO 9917-1:2007” [28]. Five groups were examined, each consisting of eight samples (*n* = 8), totaling 40 samples. The molds, groupings, sample preparation, manipulation, storage environment, and time were consistent with those used in the surface microhardness test. After 7 days, the samples were removed from the molds and allowed to air-dry for 5 min. Compressive strength was then measured by placing each sample between the plates of a universal testing machine (Zwick/Roell Z020, Ulm, Germany), applying force according to the standard at a crosshead speed of 1 mm/min. The maximum force required to break the sample was recorded, and the compressive strength (in MPa units) was calculated using the formula: *C = *4*p*/*πd*^2^, where *p* represents the maximum force applied in newton and *d* denotes the average diameter of the samples in mm.

### 2.4. FTIR Spectroscopy

Four groups were considered. Group 1 (ND-I), not dried; Group 2 (D-I), dried/directly into incubator; Group 3 (D-P), dried—stored in PBS; and Group 4 (D-W), dried—stored in distilled water. Three samples in each group (*n* = 3) and a total of 12 samples were tested. The samples were air-dried and crushed to powder using a pestle and mortar. One to 2 mg of each sample was mixed with 100 mg of potassium bromide and pressed into disks using a manual hydraulic press (Specac, Orpington, UK). The disks weighing approximately 200 mg and measuring 15 × 0.5 mm in diameter were prepared by a punch press working at 1 ton/cm^2^. These disks were then analyzed in a spectrometer machine (Bruker Tensor 27; Bruker Optics, Ettlingen, Germany). FTIR spectra were recorded in the wavenumber range of 4000 − 400 cm^−1^, in increments of 1.928 cm^−1^, at a resolution of 4 cm^−1^. The obtained results were compared with the theoretical available data.

### 2.5. Field Emission Scanning Electron Microscopy (FE-SEM)/EDS

Four groups similar to those in the FTIR test were evaluated. The samples were dried using a vacuum desiccator, mounted on separate aluminum stubs, gold-coated, and examined with a FE-SEM (Tescan Mira3, Brno, Czech Republic) equipped with EDS for the elemental analysis using Vantage software (Thermo Fisher Scientific, Waltham, MA, USA). EDS color dot map analysis was performed for each sample. For FE-SEM, settings included a working distance of 12–14 mm, SEM magnification of 10–25 *k*×, beam intensity of 10, and view field of 8–41.5 μm. For EDS and mapping, a working distance of 14.93 mm, SEM magnification of 5 *k*×, beam intensity of 17, and view field of 41.5 *μ*m were applied. An accelerating voltage of 20 kV was used for both evaluations.

### 2.6. XRD

Four groups similar to those tested in the FTIR test were evaluated. XRD patterns were recorded using an X-ray diffractometer (Philips PW 1710, Almelo, Netherlands) with Cu K*α* radiation from an X-ray generator operated at 40 kVp and 30 mA. Data were collected from a scan range of 10°–80° 2*θ* with a step size of 0.05° and 1 s time per step. Diffraction analysis software (X'Pert high score plus, Version 3; Malvern Panalytical, Malvern, UK) was used to analyze the XRD results. The obtained peaks of each group were compared and matched with the databases of the “International Center for Diffraction Data” (ICDD, Pennsylvania, USA), the “Joint Committee on Powder Diffraction Standards” (JCPDS), and the “Inorganic Crystal Structure Database” (ICSD, FIZ Karlsruhe, Germany). The relative amounts of each crystalline phase were estimated based on the size of its relevant peak.

### 2.7. Statistical Analysis

Descriptive statistics, including mean and standard deviation, were used to report the test values. Normality and homogeneity of variances were assessed using Shapiro–Wilk and Levene tests, respectively. Since the variances were equal, the independent sample *t*-test was employed to compare the setting time means between the two evaluated groups. Given the fulfillment of the conditions required for one-way ANOVA, this test was applied to compare the means of the four test groups in microhardness and compressive strength tests. Additionally, the Tukey honestly significant difference (HSD) test was used for multiple comparisons. The two-sample Kolmogorov–Smirnov test performed a pairwise comparison of the data distribution for the EDS evaluation of the major elemental constituents. A significance level of 0.05 was set for type one error, and all the analyses were conducted using SPSS software (SPSS Version 26.0; IBM, Armonk, NY, USA).

## 3. Results

### 3.1. Setting Time

The results of the setting time test are presented in Table 1. The setting time data exhibited normal distribution (Shapiro–Wilk *p* value > 0.05), and equality of variances was confirmed (*p* = 0.327, Levene statistic = 1.022). According to the independent *t*-test, Group 2 (dried) displayed a significantly lower average setting time (44.22 ± 4.41 min) compared to Group 1 (not dried) (70.55 ± 3.32 min), *p* < 0.001.

### 3.2. Microhardness

The distribution of microhardness data was confirmed to be normal (*p* > 0.05 in Shapiro–Wilk test), and the equal variance hypothesis was accepted (Levene statistic = 1.087, *p* = 0.384). The conditions for using one-way ANOVA were met. Through one-way ANOVA between groups, *F*_4,30_ = 79.68, *p* < 0.001. The results in Table 2 showed that Group 1 (not dried, placed into the incubator) exhibited the lowest Vickers microhardness (38.71 ± 2.68 kg/mm^2^), while Group 3 (dried, placed in PBS) showed the highest (78.80 ± 4.62 kg/mm^2^) Vickers microhardness. The other three groups displayed significantly higher microhardness values than Group 1 and lower than Group 3.

### 3.3. Compressive Strength

Compressive strength data demonstrated a normal distribution (Shapiro–Wilk *p* value > 0.05), and the homogeneity of variances was confirmed (Levene statistic = 0.281 and *p* = 0.888). Therefore, one-way ANOVA was applicable. According to the one-way ANOVA test, *F*_4,35_ = 25.01, *p* < 0.001. As depicted in Table 3, Group 1 (without drying, into the incubator) had the significantly lowest compressive strength (7.03 ± 1.47 MPa) compared to all other groups. The highest value was observed in Group 4 (dried, placed in distilled water) (13.79 ± 1.78 MPa), which was significantly different from all groups except Group 2 (dried, placed into the incubator).

### 3.4. FTIR

**Figure 1:**
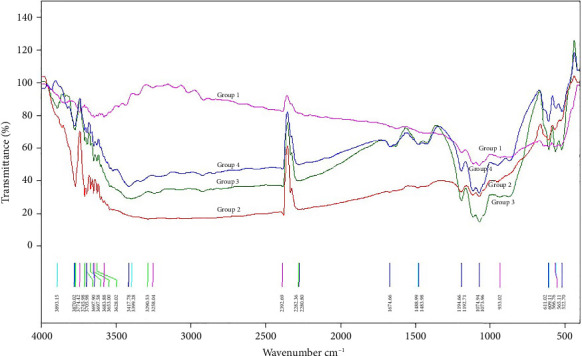
Fourier transform infrared (FTIR) spectra used to analyze chemical structural properties of four groups of calcium-enriched mixture cement samples with different placement methods and storage environments including Group 1, not dried and stored in the incubator; Group 2, dried/directly into the incubator; Group 3, dried—stored in PBS; Group 4, dried—stored in distilled water. For a better comparison, the graphs of all four groups were placed and superimposed in one figure. The vertical axis shows how much (percentage of) infrared light the substance absorbs. The horizontal axis represents the wavelength of the light. The spectra show various vibration modes at different wavelengths characteristic of various chemical/functional groups. In our study, all specimens showed absorption peaks characteristic of the (PO_4_)^3−^ group at 560–600 cm^−1^ and 1198 − 1090 cm^−1^. Additionally, vibrations attributed to Si compounds at 1050–1100 cm^−1^ were observed in the samples of all tested groups. Groups 2, 3, and 4 (dried groups) also exhibited peaks at 3780 and 3682 cm^−1^, related to the OH groups and Si(OH)_2_, respectively. Moreover, Group 3 (dried—stored in PBS) and Group 4 (dried—stored in distilled water) displayed vibration modes of (CO_3_)^2−^ ions at 1488 cm^−1^ and bands characteristic of OH groups from 3650 to 3290 cm^−1^.

Figure 1 illustrates FTIR spectra graphs of the four groups combined in one image. The unaffected peaks observed in all CEM samples included vibrations indicating (PO4)^3−^ ions and Si compounds. Notably, all specimens showed absorption peaks characteristic of the (PO_4_)^3−^ group at 560–600 cm^−1^ and 1198–1090 cm^−1^. Additionally, vibrations attributed to Si compounds at 1076 (1050–1100) cm^−1^ were observed in the samples of all tested groups.

In determining the variations in the peaks caused by manipulation methods and storage environments, further details revealed more vibration modes of extra (PO_4_)^3−^ ions around 950-965 cm^−1^ in FTIR spectra of the dried group samples (Groups 2, 3, and 4) than in the nondried group. The dried groups also exhibited peaks at 3780 and 3682 cm ^−1^, related to the OH groups and Si(OH)_2_, respectively. Moreover, Group 3 (dried—stored in PBS) and Group 4 (dried—stored in distilled water) displayed vibration modes of (CO_3_)^2−^ ions at 1488 cm^−1^ and bands characteristic of OH groups from 3650 to 3290 cm^−1^.

### 3.5. SEM

**Figure 2:**
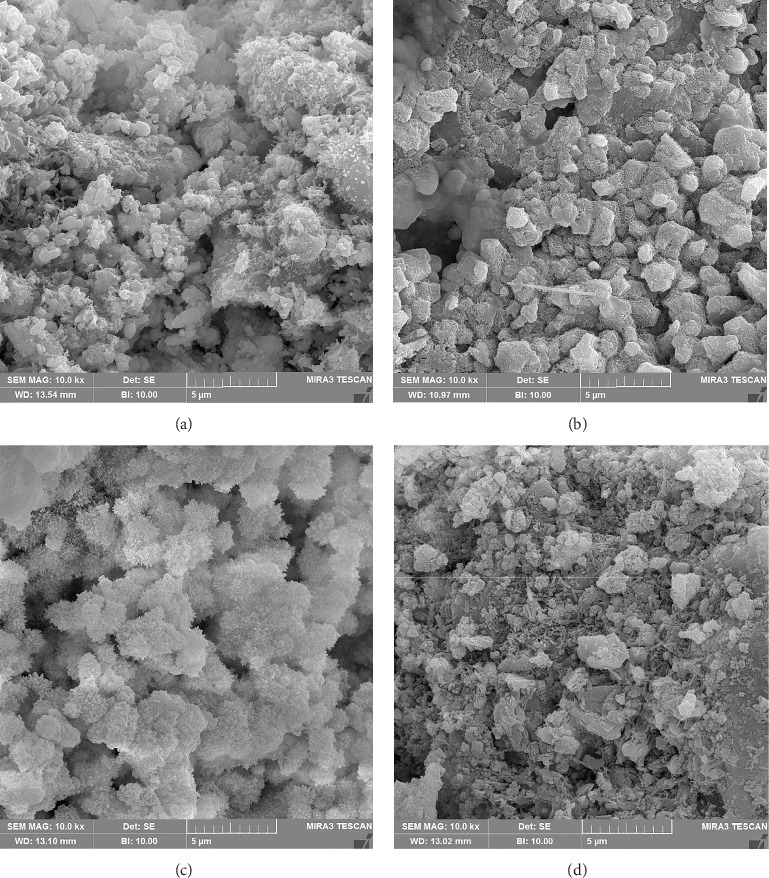
Field emission scanning electron microscopic photomicrographs of samples of CEM cement: (a) Group 1, not dried and stored in the incubator; (b) Group 2, dried/directly into the incubator; (c) Group 3, dried—stored in PBS; (d) Group 4, dried—stored in distilled water.

Figure 2 presents FE-SEM photomicrographs of different group samples. The morphological microstructure of all CEM samples demonstrated crystalline structures with coarse-grained to fine particles. Under different manipulation conditions (drying or not) and different storage environments, the crystalline structure showed a wide variety of homogeneity with a wide range of particle sizes. The SEM micrographs of Groups 1 (not dried), 2 (dried/directly into the incubator), and 4 (dried—stored in distilled water) showcased hexagonal or rectangular cubic and needle-like crystalline structures, with Group 4 exhibiting the most compact and finest crystals. In contrast, Group 3 (dried—stored in PBS) revealed a homogeneous crystal structure consisting of interconnected clusters in a globular lattice covered with compact fine crystalline structures.

### 3.6. EDS

Semiquantitative EDS evaluation revealed that the major elemental constituents in the CEM samples of all groups were oxygen (O), calcium (Ca), carbon (C), silicon (Si), sulfur (S), barium (Ba), phosphorus (P), and zirconium (Zr). Tables 4 and 5 show the mean, standard deviation (SD), and order of elemental weight percentage (W%) for different groups. The highest oxygen weight percent was found in Group 2 (dried/directly into incubator). The highest W% of calcium and phosphorus were detected in Group 3 (dried—stored in PBS). Group 4 samples (dried—stored in distilled water) had the highest W% of silicon. Despite notable differences in the aforementioned values/orders among groups, pairwise comparison using the two-sample Kolmogorov–Smirnov test displayed no significant difference between pairs of groups (exact two-tailed *p* values ranged from 0.283 to 0.980).

#### 3.6.1. EDS Dot Mapping

**Figure 3:**
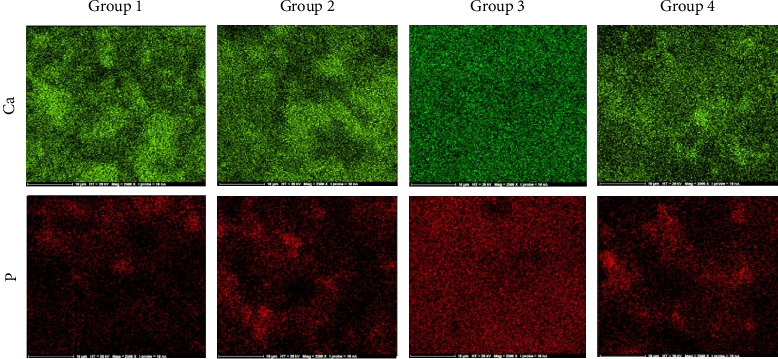
X-ray dot mapping analysis of elemental distributions of calcium (Ca) and phosphorous (P) in the four groups of CEM cement samples: Group 1, not dried and stored in the incubator; Group 2, dried/directly into the incubator; Group 3, dried—stored in PBS; Group 4, dried—stored in distilled water.

After identifying major elements and their atomic percentage concentrations, calcium and phosphorous mapping of the tested groups was performed, representing their most influential elements (Figure 3). In Group 3 (dried—stored in PBS), a compact, uniform, and homogeneous distribution of both elements in the mapping was observed, which is different from the mapping pattern of these elements in all other three groups.

### 3.7. XRD

**Figure 4:**
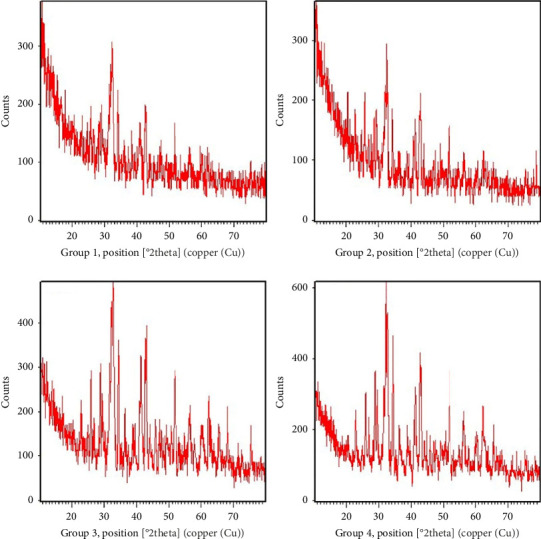
X-ray diffraction analysis of samples of CEM cement showing peaks of different crystalline phases and their relevant counts: Group 1, not dried and stored in the incubator; Group 2, dried/directly into the incubator; Group 3, dried—stored in PBS; Group 4, dried—stored in distilled water.

XRD results are illustrated in Figure 4 and Table 6. The peaks of all CEM groups matched with the reflections of tricalcium silicate (Ca_3_SiO_5_, ICDD 00-055-0738) and dicalcium silicate (Ca_2_SiO_4_, ICDD 00-70–0388), along with reflections of silicon oxide (SiO_2_, ICSD 01-076-0941) and zirconium oxide (ZrO_2_, ICDD 00-037-1484). Additionally, peaks corresponding to calcium hydroxide (CaOH_2_, ICSD 01-084-1263) and barium sulfate (BaSO_4_, JCPDS 01-080-0512) were observed in the samples of all groups.

Furthermore, XRD analysis in Group 2 (dried/directly into the incubator) revealed peaks associated with calcium carbonate (CaCO3, ICDD 00-005-0586) and broad peaks attributed to calcium silicate hydrate (3CaO.2SiO_2_.3H_2_O or C-S-H, ICDD 00-034-0002). Group 3 (dried—stored in PBS) exhibited broad strong peaks reflecting tricalcium silicate and dicalcium silicate, along with broad diffraction maxima characteristic of calcium silicate hydrate. Lastly, Group 4 (dried—stored in distilled water) showed peaks attributed to tricalcium silicate, strong peaks corresponding to dicalcium silicate, and multiple peaks associated with silicon oxide.

## 4. Discussion

Efforts to enhance the properties of dental materials are crucial for increasing the efficiency of their clinical applications [33]. The physical and mechanical characteristics of these materials are influenced by numerous factors [7], including powder-to-liquid ratio [34, 35], mixing and placement techniques [11], and storage media [12]. This study revealed that moisture absorption by the cement mixture, facilitated by dry cotton during placement, led to a reduction in setting time, while simultaneously increasing compressive strength and surface microhardness. Optimal microhardness and the highest compressive strength were achieved by immersing the cement in PBS and distilled water for 7 days, respectively.

In comparing the FTIR spectra of CEM specimens with hydroxyapatite [36], all tested CEM groups exhibited distinct peak vibrations for (PO4)^3−^ ions. Additionally, all CEM samples exhibited vibration modes associated with Si compounds [37]. Notably, silicon bands were identified in the FTIR plot of both the original gray MTA (GMTA) and white MTA (WMTA; ProRoot MTA; Dentsply Tulsa Dental, OK, USA) [38]. Additionally, bands corresponding to Si-O vibrations were observed in the FTIR spectra of Bioaggregate and Biodentine [39]. These results indicate the presence of silicon compounds and suggest potential hydroxyapatite formation, highlighting the potential bioactivity of the tested cement [40]. Moreover, all the dried samples displayed increased vibrations of (PO4)^3−^ ions compared to the undried group, implying enhanced precipitation of apatite compound and hydroxyapatite formation on the surface of dried samples [41]. Furthermore, the OH groups observed in the dried samples were attributed to water molecules, Si-OH [37], or Ca-OH [42] as reflected in the FTIR spectra of Bioaggregate and Biodentine [39]. In our study, specimens of Group 3 (dried—stored in PBS) and Group 4 (dried—stored in distilled water) demonstrated vibration modes attributed to (CO_3_)^−2^. In agreement with our results, absorption bands for (CO_3_)^−2^ vibration were also detected in GMTA, WMTA, Bioaggregate, and Biodentine, corresponding to vibration peaks observed in the CEM samples of Groups 3 and 4, which can be indicative of CaCO_3_ [38, 39]. These findings align with a previous study on ProRoot MTA (Dentsply Tulsa Dental), which demonstrated the formation of hydroxycarbonate apatite and calcium carbonate 7 days after cement storage in PBS [43].

The materials employed for root-end filling must be set rapidly to achieve the necessary strength, dimensional stability, and establish an effective seal, without displacement [44]. However, a primary drawback of using MTA in such cases is its prolonged setting time [44, 45]. We have identified a straightforward method to accelerate the setting time of this material, without the use of additional chemicals by reducing the liquid ratio in the cement mixture with the use of dry cotton pellets. Consistent with our findings regarding CEM cement, a recent study demonstrated that increasing the liquid content of Biodentine (Septodont, Saint Maur des Fossés, France) would also prolong its setting time [46].

In this investigation, all dried groups exhibited significantly greater microhardness and compressive strength compared to the nondried group, which had a higher liquid-to-powder ratio. Previous research demonstrated that reducing the liquid-to-powder ratio of CEM resulted in significantly enhanced microhardness [9]. Additionally, the lowest liquid ratio in the mixture corresponded to the highest compressive strength of CEM cement [16]. While it might be anticipated that a higher liquid proportion would improve the mechanical properties of this biomaterial by promoting the necessary aqueous reactions for setting and achieving biological properties, our findings contradicted this hypothesis. Instead, an increased liquid ratio led to a weakening in the studied mechanical properties of the cement. Similarly, an increase in the liquid dosage of Biodentine resulted in a reduction in microhardness [46]. Furthermore, the maximum microhardness was observed in samples stored in PBS, whereas the highest compressive strength was recorded in samples subjected to drying/immersion in distilled water (13.79 MPa) and drying/storage in a 90% humid incubator (13.48 MPa). The observed alternations in this study may be explained by the penetration or diffusion of water into the samples. The introduction of water into ceramic materials can strengthen their structure, leading to a phase transformation in their crystalline phase [47].

In the current XRD analysis, the common peaks across all groups corresponded to reflections of tricalcium silicate, dicalcium silicate, calcium hydroxide, zirconium oxide, and barium sulfate. Additionally, samples of the dried groups (Groups 2, 3, and 4) exhibited peaks associated with calcium carbonate and calcium silicate hydrate. A recent study on CBS cements [20], which evaluated CEM specimens stored in PBS for 1 week, identified reflections of tricalcium silicate, dicalcium silicate, calcium hydroxide, calcium carbonate, and barium sulfate, consistent with our findings. However, that study did not observe reflections of calcium silicate hydrate and zirconium oxide [20]. Another evaluation of the crystalline phases of CBS cement using Rietveld-XRD analysis showed that the diffractogram of hydrated CEM cement included tricalcium silicate, dicalcium silicate, and barium sulfate, similar to our study. Additionally, it reported peaks of calcium sulfoaluminate (ettringite), a product of Portland cement hydration, which was not observed in our study [48]. These findings suggest that variations in the amount and method of cement hydration, manipulation, and storage conditions can lead to the formation of different crystalline phases and structures, which should be considered in clinical applications.

In our investigation, samples from Groups 3 and 4, which were stored in PBS and distilled water, respectively, displayed the most prominent peaks of tricalcium silicate and dicalcium silicate. Tricalcium silicate, a key component of CSB cements such as MTA, serves as the primary raw material for paste preparation in Portland cement. When exposed to simulated body fluid, tricalcium silicate exhibited the ability to induce apatite formation [49, 50]. Dicalcium silicate, which is also present in Portland cement, refractory materials, and MTA, is another major component of CSB cements [51]. The presence of tricalcium silicate and dicalcium silicate as primary constituents in CSB cements, such as MTA and CEM, contributes to their exceptional sealing ability [49]. The hydration reaction of the CSB cements initiates with the dissolution of tricalcium silicate in water, followed by the precipitation of calcium ions, silicate gel, and hydroxide (OH^−^) ions [49]. In our study, samples stored in distilled water exhibited the highest counts of tricalcium and dicalcium silicates, silicon oxides, and calcium hydroxide reflections. The improved compressive strengths observed in this group may be ascribed to enhanced hydration reaction and increased formation of these compounds. Furthermore, the drying of the cement mixture combined with its immersion in PBS for 7 days led to increased reflection/formation of tricalcium silicate and calcium silicate hydrate, resulted in the most significant increase in the cement's microhardness. The formation of more calcium silicate hydrates and portlandite calcium hydroxide as major final products of the hydration reaction in CSB cements may contribute to the development of a more compact surface microstructure, as observed in SEM evaluation and elemental mapping of specimens from Group 3 (dried—stored in PBS) in this study [49].

The FE-SEM analysis of all CEM samples revealed crystalline structures ranging from coarse-grained to fine particles. Through the application of various placement methods and storage media in this study, hexagonal, rectangular cubic, globular, and needle-like/tag-like crystalline particles with different diverse patterns of fineness and homogeneity were observed. These findings are consistent with previous research on CBS cements, demonstrated that all CSB cements consist of globular/spherical, cubic, and/or needle-like crystals [20, 24]. Nevertheless, a recent study reported hexagonal crystals after 1 week of contact between CEM specimens and PBS [20]. Our study revealed a homogeneous globular crystalline microstructure covered with fine crystals after drying and 1 week of storage in PBS (Group 3). This distinct microstructural appearance may be attributed to variations in sample preparation and the drying process. Homogeneous distribution of smaller sized crystals was also observed in the SEM analysis of Biodentine [52]. Furthermore, crystalline structures indicative of apatite formation on the surface of other CBS cements after immersion in PBS have been observed, becoming more uniform and smooth with increased immersion time [53]. Moreover, these compact, fine-sized crystalline tags have the potential to increase the contact surface of the cement with adjacent dentin. This enhancement may lead to better adaptation to dentinal tubules, an increased surface area, and heightened reactivity of cement particles in forming calcium hydroxide and calcium silicate hydrate phases, similar to observations in ProRoot MTA (Dentsply Tulsa Dental, Tulsa, OK, USA) [24, 54].

EDS is a semiquantitative technique with an accuracy of approximately ±2% for major constituents [26]. The preliminary EDS analysis of CEM cement revealed CaO > H2O-CO_2_ > SO_3_ > P_2_O_5_ > SiO_2_ as major components, along with Al_2_O_3_, Na_2_O, MgO, and Cl as minor components [55]. In this study, the principal elemental constituents of the cement were oxygen (O), calcium (Ca), carbon (C), silicon (Si), sulfur (S), barium (Ba), phosphorus (P), and zirconium (Zr), with varying weight percentages depending on the placement method and environmental conditions. Although the differences in the weight percentages of the major elements among the various CEM groups were not statistically significant, the Group 3 samples (dried—stored in PBS) exhibited the highest weight percentages of calcium and phosphorous, along with the lowest Ca/P molar ratio, following this order of weight percentages: O > Ca > P > Zr > Ba > C > Si > S. In contrast, a previous EDS evaluation of CEM specimens exposed to PBS for 7 days reported a different order of elements weight percentages: Ca > O > Ba > Zn > P > S > Si > C, demonstrating a higher percentage of calcium and a significantly lower percentage of phosphorous [20]. These variations may be attributed to differences in sample preparation and element probes, leading to distinct findings. Furthermore, the EDX results of Biodentine exposed to PBS identified carbon, oxygen, calcium, zirconium, chlorine, silicon, and sodium as its major elemental constituents [20], while EDX analysis of Bioaggregate indicated the presence of calcium, silicon, oxygen, phosphorous, and zirconium [39]. Additionally, elemental dot mapping of the samples in Group 3 revealed a compact and uniform distribution of calcium (Ca) and phosphorus (P), presenting a markedly differing from the distributions observed in the other three groups. This observation might be indicative of apatite formation in CEM cement upon contact with PBS and similar tissue fluids. Previous studies have shown that the interaction between calcium and phosphorus ions during the setting of this cement can suggest the formation of hydroxyapatite [55, 56].

In similar studies, various grit sandpapers have been utilized for microhardness testing. In the present study, however, a 1200-grit sandpaper was delicately applied to avoid removing the surface layer, thereby allowing for a precise assessment of the effects of the surrounding environment (i.e., incubator moisture, PBS, distilled water) in direct contact with the layer. It is important to note that a limitation of the current investigation is its restriction to an in vitro setting. Additionally, safely removing the set samples intact from their metal molds posed a challenge.

In investigations of various CSB cements, multiple aspects of their bioactivity have been explored, including odontogenic differentiation and the adhesion behavior of human dental pulp stem cells (DPSCs) on different cements before and after setting [57–59]. On both MTA and CEM, DPSCs exhibited adhesion, proliferation, and differentiation. Notably, these two CSB cements induce different gene expressions and growth factor release [59]. Moreover, tests conducted on CEM cement samples at the root end revealed that both set and unset samples exhibited cellular adhesion and favorable clinical outcomes [60]. Overall, CEM demonstrated the highest adhesion capacity for stem cells compared to other biomaterials used in endodontics [58]. Future studies could delve into cell adhesion to samples of this cement with different placement and manipulation methods, and storage environments.

## 5. Conclusions

This in vitro study has provided important insights into the manipulation and storage effects on the properties of CEM cement. The findings highlight the role of incremental drying during placement, revealing a notable reduction in setting time. Furthermore, the study demonstrates that drying and subsequent storage in PBS or distilled water for 1 week contribute to enhanced microhardness and compressive strength. The increased diffraction peaks of tricalcium and dicalcium silicates further support these mechanical improvements.

A noteworthy observation is the highest calcium and phosphorous weight percentages observed after drying and preservation in PBS, resulting in a uniform and compact distribution in the elemental mapping analyses. Additionally, FTIR spectra revealed vibration modes of (PO_4_)^3−^ ions and Si compounds across all groups. Samples exposed to PBS or distilled water exhibited increased vibrations of (PO_4_)^3−^, (CO_3_)^2−^ ions, and O-H groups, indicating a potential for hydroxyapatite formation on the cement surface and suggesting possible bioactivity.

## Figures and Tables

**Table 1:** Descriptive analysis of setting time data (in minutes) and *t*-test comparison.

Groups	Mean (±SD)	*T*-test for equality of means			
		*t*	df	*p* value (2-tailed)	Mean difference
1. Not dried	70.55 (±3.32)	14.31	16	< 0.001⁣^∗^	26.33
2. Dried	44.22 (±4.41)				

Abbreviation: SD = standard deviation.

⁣^∗^The significance level was set at 0.05.

**Table 2:** Descriptive analysis of microhardness data (in kg/mm^2^ unit).

Group	*n*	Mean	SD
1. Not dried	7	38.71^a^	2.68
2. Dried to incubator	7	67.66^be^	4.88
3. Dried to PBS	7	78.80^c^	4.62
4. Dried to distilled water	7	70.47^de^	4.25
5. Dried to 1/2 PBS	7	69.38^de^	5.70

*Note:* Different lowercase letters indicate significant differences in microhardness through post hoc test/Tukey HSD post hoc test (*p* < 0.05), in Groups 1–5.

**Table 3:** Descriptive analysis of compressive strength data (in MPa).

Group	*n*	Mean	SD
1. Not dried	8	7.03^a^	1.47
2. Dried to incubator	8	13.49^bcd^	1.64
3. Dried to PBS	8	11.29^ce^	1.26
4. Dried to distilled water	8	13.79^d^	1.78
5. Dried to 1/2 PBS	8	10.43^e^	1.52

*Note:* Different lowercase letters indicate significant differences in compressive strength through post hoc test/Tukey HSD (*p* < 0.05), in Groups 1–5.

**Table 4:** Results of EDS semiquantitative analysis of major elemental constituents' weight percentage (W%) of different tested groups of CEM cement.

Elements	W% mean (±SD)			
	Group 1, not dried to incubator	Group 2, dried/directly to incubator	Group 3, dried—stored in PBS	Group 4, dried—stored in distilled water
Oxygen	40.19 (±5.39)	47.37 (±4.14)	40.53 (±5.67)	40.75 (±3.75)
Calcium	29.51 (±1.14)	35.42 (±3.32)	36.68 (±5.14)	31.56 (±1.71)
Carbon	8.46 (±4.84)	4.80 (±3.16)	2.42 (±0.86)	2.49 (±0.28)
Silicon	5.53 (±1.65)	2.63 (±0.27)	1.82 (±0.18)	6.29 (±2.46)
Barium	5.48 (±0.15)	3.17 (±1.68)	2.71 (±1.60)	5.52 (±0.10)
Sulfur	4.84 (±0.97)	2.53 (±0.97)	1.43 (±0.62)	3.68 (±1.19)
Phosphorous	4.30 (±1.80)	4.98 (±2.78)	9.69 (±2.44)	7.90 (±2.08)
Zirconium	1.67 (±0.23)	1.41 (±0.60)	4.69 (±1.19)	1.78 (±0.50)

Abbreviation: SD = standard deviation.

**Table 5:** Order of the weight percentage of the major elemental constituents of different tested groups of CEM cement.

Group	Order of weight percentage of elements
1. Not dried to incubator	O > Ca > C > Si > Ba > S > P > Zr
2. Dried to incubator	O > Ca > P > C > Ba > Si > S > Zr
3. Dried to PBS	O > Ca > P > Zr > Ba > C > Si > S
4. Dried to distilled water	O > Ca > P > Si > Ba > S > C > Zr

Abbreviations: Ba = barium, C = carbon, Ca = calcium, O = oxygen, P = phosphorus, S = sulfur, Si = silicon, Zr = zirconium.

**Table 6:** X-ray diffraction data of the samples of CEM cement showing important matched reflection peaks of angles 2*θ*; Group 1, not dried and stored in the incubator; Group 2, dried/directly into the incubator; Group 3, dried—stored in PBS; Group 4, dried—stored in distilled water.

Peaks of angles 2 *θ*	Matched compound							
	Tricalcium silicate	Dicalcium silicate	Calcium hydroxide	Barium sulfate	Silicon oxide	Zirconium oxide	Calcium carbonate	Calcium silicate hydrate
Group 1	32.34	31.88	18.37	28.64	23.05	28.39	—	—
Group 2	32.53–32.68	31.89 and 32.09	18.04 and 18.59	42.97	23.59	28.64	29.19–29.43	15.49–16.75
Group 3	32.64–32.99, and 33.02–33.24	31.27–31.99	18.14	42.68 and 43.03	26.09	28.27 and 28.84	29.34 and 29.49	16.71, 23.59, and 29.18
Group 4	32.54–32.69	34.26–34.42	18.53 and 18.79	28.84 and 42.69	23.15, 23.54, 23.79, and 24.78	28.78 and 28.89	29.54	29.49 and 51.49

## Data Availability

The data that support the findings of this study are available from the first author upon reasonable request.

## Nomenclature

CEM: Calcium-enriched mixture
ND-I: Not dried, placed directly in an incubator
D-I: Dried, placed directly in an incubator
PBS: Phosphate-buffered saline
D-P: Dried samples stored in PBS
D-W: Dried samples stored in distilled water
FTIR: Fourier transform infrared spectroscopy
XRD: X-ray diffraction
SEM: Scanning electron microscopy
EDS: Energy-dispersive X-ray spectroscopy
ANOVA: Analysis of variance
Tukey HSD test: Tukey honestly significant difference test
CBS: Calcium silicate–based
MTA: Mineral trioxide aggregate
RIDS: Research Institute for Dental Sciences
FE-SEM: Field emission scanning electron microscopy
ICDD: International Center for Diffraction Data
JCPDS: Joint Committee on Powder Diffraction Standards
ICSD: Inorganic Crystal Structure Database
SD: Standard deviation
CI: Confidence interval
O: Oxygen
Ca: Calcium
C: Carbon
Si: Silicon
S: Sulfur
Ba: Barium
P: Phosphorus
Zr: Zirconium
W%: Weight percentage
DPSCs: Dental pulp stem cells
